# *De novo* transcriptome assembly of fluorine accumulator tea plant *Camellia sinensis* with fluoride treatments

**DOI:** 10.1038/sdata.2018.194

**Published:** 2018-09-25

**Authors:** Qing-Sheng Li, Xu-Min Li, Ru-Ying Qiao, En-Hui Shen, Xiao-Ming Lin, Jian-Liang Lu, Jian-Hui Ye, Yue-Rong Liang, Xin-Qiang Zheng

**Affiliations:** 1 Tea Research Institute, College of Agriculture and Biotechnology, Zhejiang University, Hangzhou, 310058, China; 2 Tea Department, Wuzhou University, Wuzhou, 543000, China; 3 Institute of Crop Science & Institute of Bioinformatics, College of Agriculture and Biotechnology, Zhejiang University, Hangzhou, 310058, China

**Keywords:** Plant molecular biology, Agricultural genetics, RNA sequencing

## Abstract

Tea plant (*Camellia sinensis*) is a typical fluoride (F) hyperaccumulator enriching most F in old leaves. There is association between the risk of fluorosis and excessive consumption of teas prepared using the old leaves. It is meaningful to develop methods for controlling F levels in tea leaves. We generated a comprehensive RNA-seq dataset from tea plants grown at various F levels for different durations by hydroponics, aiming at providing information on mechanism of F metabolism in tea plant. Besides raw reads of the RNA-seq dataset, we present assembled unigenes and aligned unigenes with annotations versus the Gene Ontology (GO) databases, Kyoto Encyclopaedia of Genes and Genomes (KEGG) databases, and Nonredundant (Nr) protein databases with low e-values. 69,488 unigenes were obtained in total, in which 40,894 were given Nr annotations.

## Background & Summary

Tea plants accumulate abundant fluoride (F) from environments without toxicity^1–3^. The F concentration in mature tea leaves was up to 2800 mg/kg^4^. In general, tea brewing leaches 24–83% of total F into infusions^4^. An adult drinking five cups of tea per day would intake 8.0–303% F content of the Polish SAI (Safe and Adequate Daily Intake)^5^. Long-term consumption of brick teas with excess F would increase the risk of dental and skeletal fluorosis^6,7^.

Fluoride uptake by tea plant is highly related to Al, Ca and anion channels. Adding Al to the soil or hydroponic solution increased F accumulation in tea plant but had no effect on Al accumulation^8^. Tea plant might absorb F through a F-Al complex^9^. Endogenous Ca^2+^ and CaM played an important role in F accumulation when there was Al^3+^ in the hydroponic solution^10^. The addition of Ca^2+^ decreased the passages in cell wall or membrane, resulting in reduction of F uptake^11^. F uptake by tea plants was related to anion channel, because anionic channel inhibitor NPPB or DIDS could reduce F absorption^12^.

For non-model organisms with limited information of genome, ribonucleic acid sequencing (RNA-Seq) is an efficient approach to transcriptome profiling^13–15^. In this study, RNA-Seq was tested on tea leaf samples from hydroponic tea plants grown at three levels of F for two growing duration, with two biological replicates and control. Based on 10 cDNA libraries, a *de novo* assembled transcriptome was generated using all F treatments and control samples. After *de novo* assembly, total 69,488 unigenes were obtained with N50 of 869 bp. The unigene dataset can help explore the potential genes towards F metabolism in tea plant.

## Methods

### Experiment design

**Figure 1:**
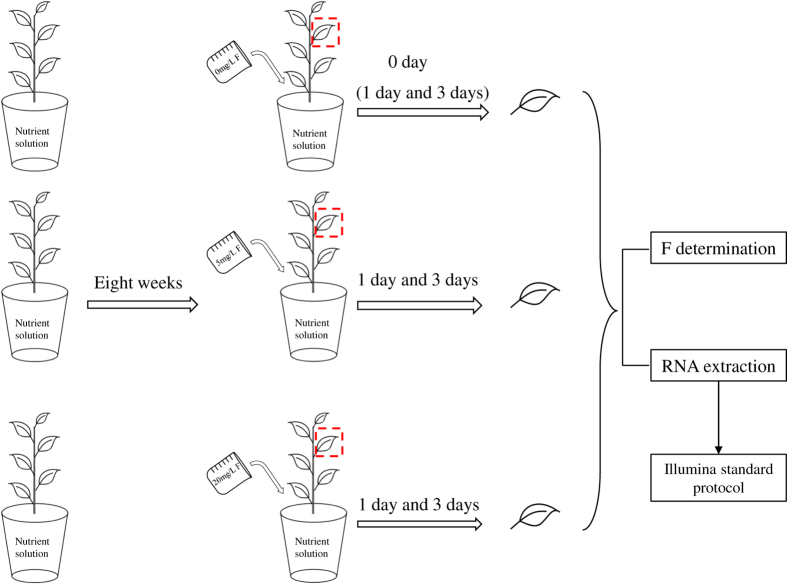
Experiment design and sampling standard. Sampling on 0 day under 0 mg/L F treatment was used as control (tabbed as Ftea-CK), and sampling on 1 and 3 days under 0 mg/L F treatment were used for F determination; Ftea-S5-1: 5 mg/L F treatment for 1 day; Ftea-S5-3: 5 mg/L F treatment for 3 days; Ftea-S20-1: 20 mg/L F treatment for 1 day; Ftea-S20-3: 20 mg/L F treatment for 3 days. F levels in all samples were measured and the *de novo* assembled transcriptome was based on Ftea-CK, Ftea-S5-1, Ftea-S5-3, Ftea-S20-1, and Ftea-S20-3 with biological replicates.

Two-year-old tea cuttings of *Camellia sinensis* cv. ‘JK2’ were cultured by hydroponic method in a climate chamber at 25±2 °C, 70±10% relative humidity and 12 h light/12 h dark^16^. The nutrient solution was renewed weekly with formula as below: 100.05 mg/L NH_4_NO_3_, 34.68 mg/L KH_2_PO_4_, 1.64 mg/L K_2_HPO_4_, 2.15 mg/L CaSO_4_·2H_2_O, 49.00 mg/L MgSO_4_·7H_2_O, 33.32 mg/L Al_2_(SO_4_)_3_·10H_2_O, 0.28 mg/L FeSO_4_·7H_2_O, 14.21 mg/L Na_2_SiO_3_·9H_2_O, 5.00 mg/L H_3_BO_3_, 3.00 mg/L MnSO_4_, 0.44 mg/L ZnSO_4_·7H_2_O, 0.16 mg/L CuSO_4_·5H_2_O, 0.16 mg/L Na_2_MoO_4_·2H_2_O, and the pH of the solution was adjusted to 4.8–5.2 by 0.1 mol/L HCl or 0.1 mol/L NaOH^16^. After eight weeks acclimation in the nutrient solution, the tea plants were treated with three levels of F (0, 5, 20 mg/L). Third leaf from apical bud with biological replicates were sampled for F content analysis and RNA extraction on the day before F treatment using 0 mg/L group as control, and 5 mg/L, 20 mg/L combined with 1^st^, 3^rd^ day as treatment groups. The labels of F treatment were as below, sampling before F treatment from 0 mg/L F group was used as control (tabbed as Ftea-CK), 5 mg/L F for 1 day (tabbed as Ftea-S5-1), 5 mg/L F for 3 days (tabbed as Ftea-S5-3), 20 mg/L F for 1 day (tabbed as Ftea-S20-1), 20 mg/L F for 3 days (tabbed as Ftea-S20-3). The experiment design and the sampling standard were illustrated in Fig. 1.

### Fluoride determination

F contents in tea samples were determined using F ion selective electrode (Shanghai Ruosull Technology Co., Ltd., Shanghai China) mainly following the method described by Stevens *et al*.^17^. To inactivate enzymes in tea leaves, we added a pretreatment of F determination by microwaving tea leaves for 60 s. Then the leaves were dried at 120 °C for 30 min and at 75 °C for 3 h to 48 h until the weights of the leaves remained unchanged. Dry samples (0.15 g) were accurately measured into conical flasks with 20 mL boiling water for 30 min and shook up per 10 min. Then the solutions were transferred into 50 mL volumetric flask with TISAB solution (3 mol/L sodium acetate: 0.75 mol/L sodium citrate=1:1 v/v) and metered the solution to 50 mL with ddH_2_O. Each solution was measured by F ion selective electrode until the change in mV was less than 0.2 mV/min. The standard curve was constructed by NaF (AR grade, dried at 105 °C for 2 h). All F concentrations of samples were calculated by direct calibration from the standard curve. The reclaim rate of the added F was 95.0–99.3%, with coefficient of variation 2.1%. The results of F contents in different tea samples were listed in Table 1.

### RNA extraction

Total RNA was extracted using an RNAprep pure plant kit special for plants with high content of polysaccharide or polyphenols (TIANGEN Biotech Co., Ltd., Beijing, China). The quality and quantity of extracted RNA were measured by agarose gel electrophoresis and Nanodrop 2000 (Quawell Technology, Inc., San Jose, USA). The extracted RNA samples were stored at −80 °C.

### Library construction and transcriptome sequencing

**Figure 2:**
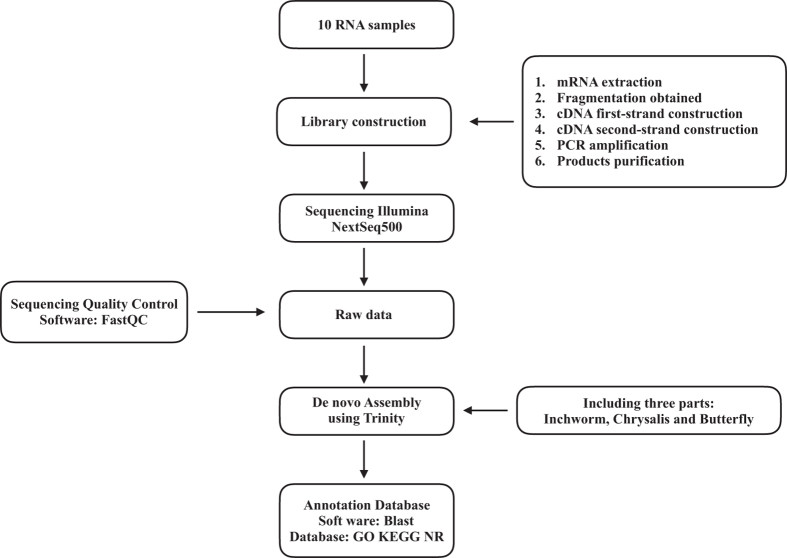
Schematic overview of the study. We collected 5 samples with replicates (third leaf from bud) including control and various fluoride treatments. After cDNA construction, Illumina NextSeq500 was used for sequencing in 150 bp paired-end (PE) reads. Trinity was used for clean reads *de novo* assembly and BlastX was used searching against GO, KEGG and NR databases.

Three μg total RNA of each sample was used for cDNA library construction using TruSeq Stranded mRNA LT Sample Prep Kit (Illumina, San Diego, CA, USA). The mRNA was extracted from total RNA by oligo (dT)-attached magnetic beads. A cDNA library was generated before Next-Generation Sequencing (NGS) in five steps: (1) The mRNA was fragmented using divalent cations under elevated temperature in an Illumina proprietary fragmentation buffer, with mRNA fragment length ranging from 200 to 300 bp; (2) First-strand cDNA was synthesized by random oligonucleotides and SuperScript II using the mRNA fragments as template. (3) Second-strand cDNA was synthesized in a mixture of buffer, dNTPs, RNase H, and DNA polymerase I, in which thymine (T) was replaced by uracil (U) so as to generate strand-specific library. (4) DNA fragments with ligated adaptor molecules on both ends were selectively enriched using Illumina PCR Primer Cocktail in a 15 cycle PCR reaction. After PCR amplification of the cDNA library, the libraries between 300–400 bp were chosen for next step. (5) Products were purified (AMPure XP system) and quantified using the Agilent high sensitivity DNA assay on a Bioanalyzer 2100 system (Agilent). Finally, Illumina NextSeq500 was performed to generate 2×150 bp paired-end (PE) reads. The major process of the study was listed in Fig. 2.

### *De novo* assembly and annotation

Before assembly, the reads with low quality were removed and adapters were filtered with Cutadapt (Version 1.2.1)^18^. Clean reads were pooled and RNA-Seq *de novo* assembly was carried out using Trinity, including assembling the reads into contigs by Inchworm, clustering the contigs to generate De Brujin Graph (DBG) by Chrysalis, and obtaining transcripts based on DBG^19^. The fixed default *K-mer* value was 25. Details of contigs and unigenes were listed in Table 2. The obtained unigenes were annotated by conducting a local BLASTx search. To classify the functions of contigs, GO annotation was performed using Blast2GO software^20^, and KEGG orthology and pathway annotations were obtained by KAAS (KEGG Automatic Annotation Server). These methods are expanded versions of descriptions in our related work^16^.

## Data Records

The raw data (Data Citation 1 and Table 3) was deposited in the NCBI Sequence Read Archive. Each accession has two replicates. The assembled unigenes have been deposited at GenBank (Data Citation 2).

## Technical Validation

To first control the sequencing quality, we compared total reads and total bases of each sample to ensure the amounts stood the same magnitude. The Q20, base content, GC content and sequence base quality were then determined using FastQC (http://www.bioinformatics.babraham.ac.uk/projects/fastqc). In order to comprehensively cover the transcriptome of *Camellia sinensis*, ten libraries of control and experimental groups were sequenced and assembled. A total of 270 573 372 raw reads were generated. 268 766 730 clean reads were obtained for *de novo* assembly after filtration, with clean reads rate being up to 99.33% (Table 4). 219 018 contigs and 69 488 unigenes were obtained (Table 2).

**Figure 3:**
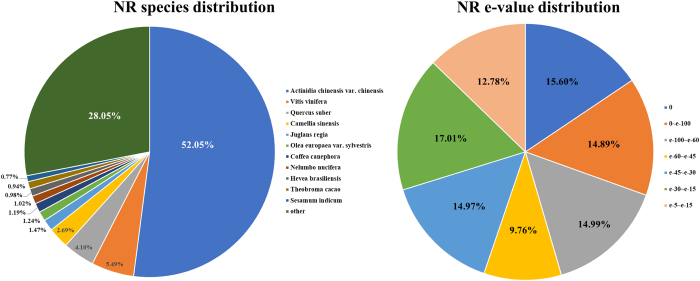
Distribution of NR e-value and species.

**Figure 4:**
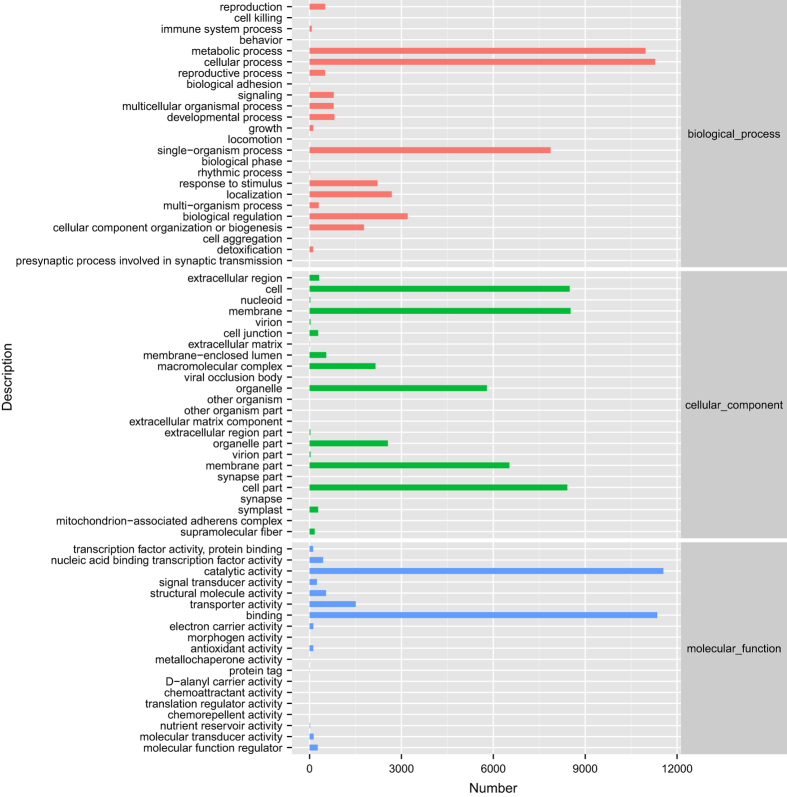
GO functional annotations of *Camellia sinensis* transcriptome. Bars represent the numbers of unigenes assigned into GO sub-categories of three main categories: biological process, cellular component and molecular function. Within the biological process category, “cellular process”, “metabolic process”, and “single-organism process” were the most abundant sub-categories. In cellular component category, the predominant portion of unigenes represented “membrane”, “cell” and “cell part” followed by “membrane part” and “organelle”. Under the molecular function category, “catalytic activity” and “binding” sub-categories were the major proportions of unigenes.

**Figure 5:**
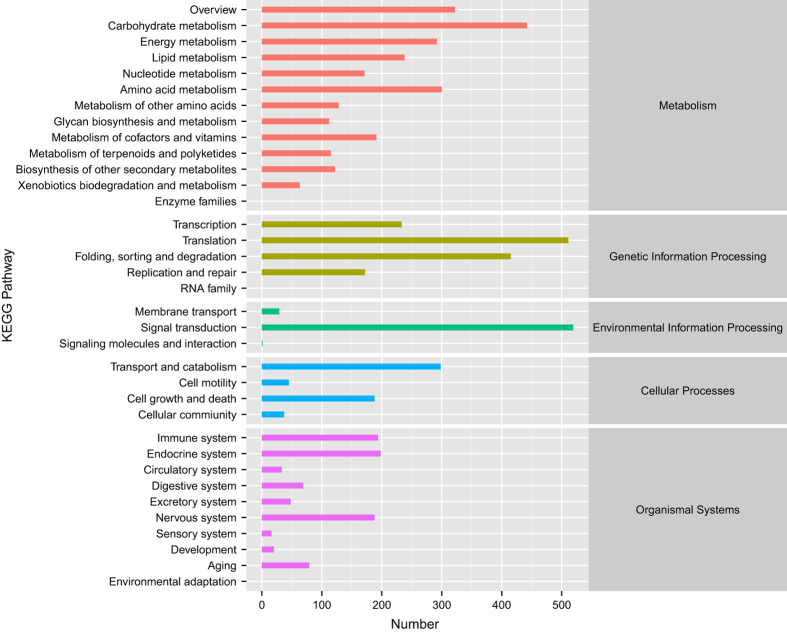
KEGG pathway annotations of *Camellia sinensis* transcriptome. Bars represent the numbers of unigenes clustered into KEGG Orthology (KO) hierarchies. “Signal transduction”, “translation” and “carbohydrate metabolism” were the most abundant categories in KO hierarchies. Certain category as “transport and catabolism” was highly related to experimental conditions.

Functional annotations were obtained by sequence based alignments performed by blast search (BLASTx) against the non-redundant protein database (Nr). The Nr species distribution and e-value distribution (Fig. 3) revealed that 55.24% of unigenes with hits had a strong homology with the sequences available in the Nr protein database (e-value < e^−45^). The distribution of Nr species was revealed that the majority of *Camellia sinensis* unigenes showed the highest homology with *Actinidia chinensis* var*. chinensis* (52.05%), then *Vitis vinifera* (5.49%), *Quercus suber* (4.10%), *Camellia sinensis* (2.69%), *Juglans regia* (1.47%), *Olea europaea* var*. sylvestris* (1.24%), *Coffea canephora* (1.19%), *Nelumbo nucifera* (1.02%), *Hevea brasiliensis* (0.98%), *Theobroma cacao* (0.94%), *Sesamum indicum* (0.77%), and others (28.05%). Because of limited Nr annotations of *Camellia sinensis*, only 2.69% of the unigenes had Nr annotations against *Camellia sinensis*. However, 93.19% (64 756 out of 69 488) of all unigenes could mapped on newly published tea genome database using tophit software. Unigenes unable to mapped on tea genome database and predicted fusion genes information were offered in appendix (Supplementary Table 1 and Supplementary Table 2). The major distribution of GO annotations was listed in Fig. 4, based on the Blast2GO software analysis. GO database includes three main categories: biological process, cellular component and molecular function. Within the biological process category, “cellular process”, “metabolic process”, and “single-organism process” were the most abundant sub-categories. In cellular component category, the predominant portion of unigenes represented “membrane”, “cell” and “cell part” followed by “membrane part” and “organelle”. Under the molecular function category, “catalytic activity” and “binding” sub-categories were the major proportions of unigenes. The distribution of KEGG pathways annotations was shown in Fig. 5. Sub-categories as “signal transduction”, “translation” and “carbohydrate metabolism” were the most abundant categories in KO hierarchies. Besides, certain category as “transport and catabolism” was highly related to experimental conditions. The summary of databases annotation was listed in Table 5.

## Additional information

**How to cite this article**: Li, Q.S. *et al*. *De novo* transcriptome assembly of fluorine accumulator tea plant *Camellia sinensis* with fluoride treatments. *Sci. Data*. 5:180194 doi: 10.1038/sdata.2018.194 (2018).

**Publisher’s note**: Springer Nature remains neutral with regard to jurisdictional claims in published maps and institutional affiliations.

## Supplementary Material



Supplementary Table 1

Supplementary Table 2

## Figures and Tables

**Table 1:** The results of fluoride treatment on F levels in tea leaves.

**Time (d)**	**F level (mg/kg)**		
	**Ftea-CK**	**Ftea-S5**	**Ftea-S20**
1	180.6 ± 1.3	265.5 ± 4.5	305.4 ± 3.5
3	179.4 ± 1.2	376.7 ± 2.4	513.3 ± 4.2
Time (d): F treating duration on tea cuttings. F level (mg/kg): F contents in tea samples. Ftea-CK: tea cuttings under 0 mg/L F concentration in hydroponic solutions. Ftea-S5: tea cuttings under 5 mg/L F concentration in hydroponic solutions. Ftea-S20: tea cuttings under 20 mg/L F concentration in hydroponic solutions.			

**Table 2:** Summary of contigs and unigenes.

	**Contig**	**Unigene**
Total Length (bp)	6,66,34,674	4,08,14,856
Sequence Number	2,19,018	69,488
Max. Length (bp)	7,149	6,325
Mean Length (bp)	304.2	587.4
N50 (bp)	412	869
N50 Sequence No.	35,411	13,326
N90 (bp)	131	250
N90 Sequence No.	1,60,691	51,233
GC%	45.36	43.75
Total Length (bp): the sum length of all contigs or unigenes. Sequence Number: the number of assembled contigs or unigenes. Max length: the length of longest contig or unigene. Mean length: the average length of contigs or unigenes. N50 (bp): arranging all sequences from longest to shortest, then adding the sequences with permutation. When the sum of the sequences reaches to 50% of total length, the length of last sequence is N50 (bp). N50 Sequence NO: the number of sequences longer than N50. N90 (bp): arranging all sequences from longest to shortest, then adding the sequences with permutation. When the sum of the sequences reaches to 90% of total length, the length of last sequence is N90 (bp). N90 Sequence NO: the number of sequences longer than N90. GC%: the percentage of GC in all bases.		

**Table 3:** Summary of samples submitted to NCBI Sequence Read Archive.

**Sample No**	**Accession**	**SRA**	**BioSample**
1	Ftea-CK rep1	SRR6189369	SAMN07811449
2	Ftea-CK rep2	SRR6189370	SAMN07811450
3	Ftea-S5-1 rep1	SRR6189371	SAMN07811451
4	Ftea-S5-1 rep2	SRR6189372	SAMN07811452
5	Ftea-S5-3 rep1	SRR6189365	SAMN07811453
6	Ftea-S5-3 rep2	SRR6189366	SAMN07811454
7	Ftea-S20-1 rep1	SRR6189367	SAMN07811455
8	Ftea-S20-1 rep2	SRR6189368	SAMN07811456
9	Ftea-S20-3 rep1	SRR6189373	SAMN07811457
10	Ftea-S20-3 rep2	SRR6189374	SAMN07811458
Ftea-CK: sampling before F treatment from 0 mg/L group and used as control; Ftea-S5-1: 5 mg/L F treatment for 1 day; Ftea-S5-3: 5 mg/L F treatment for 3 days; Ftea-S20-1: 20 mg/L F treatment for 1 day; Ftea-S20-3: 20 mg/L F treatment for 3 days. Rep 1 and rep 2 means biological replicates.			

**Table 4:** Raw data and clean reads for each accession.

**Sample**	**Raw Reads**	**Clean Reads**	**Clean Reads%**
Ftea-CK rep1	2,24,98,116	2,23,53,496	99.36%
Ftea-CK rep2	2,81,05,672	2,79,17,452	99.33%
Ftea-S5-1 rep1	2,39,92,856	2,38,27,212	99.31%
Ftea-S5-1 rep2	3,17,59,734	3,15,47,722	99.33%
Ftea-S5-3 rep1	2,57,44,530	2,55,70,978	99.33%
Ftea-S5-3 rep2	2,71,29,926	2,69,54,244	99.35%
Ftea-S20-1 rep1	2,54,18,168	2,52,49,726	99.34%
Ftea-S20-1 rep2	3,18,76,778	3,16,67,652	99.34%
Ftea-S20-3 rep1	2,67,54,336	2,65,64,954	99.29%
Ftea-S20-3 rep2	2,72,93,256	2,71,13,294	99.34%
Total	27,05,73,372	26,87,66,730	99.33%
Raw Reads: reads from next-generation sequencer. Clean Reads: high quality reads after eliminating contaminations and adaptors. Clean Reads%: the percentage of clean reads.			

**Table 5:** Summary of annotations on different databases.

**Database**	**Number**	**Percentage%**
NR	40,894	58.85
GO	23,260	33.47
KEGG	6,212	8.94
